# Methicillin resistant *Staphylococcus Aureus* in emergency department patients in the United Arab Emirates

**DOI:** 10.1186/s12873-018-0164-7

**Published:** 2018-05-15

**Authors:** Muna Al Jalaf, Hanan Fadali, Rasha Alanee, Firas Najjar, Zulfa Al Deesi, Rania M. Seliem, Eric J. Nilles

**Affiliations:** 1 Department of Emergency Medicine, Rashid Hospital and Trauma Centre, Dubai Health Authority, Dubai, UAE; 2 Laboratory Medicine Department, Rashid Hospital and Trauma Centre, Dubai Health Authority, Dubai, UAE; 3 Department of Emergency Medicine, Brigham & Women’s Hospital, Boston, USA; 4 Harvard Medical School, Boston, USA

**Keywords:** Community-associated methicillin resistant *staphylococcus aureus*, MRSA, United Arab Emirates, Middle East, Emergency, SSTI, Abscess, Skin infection, Antibiotic resistance

## Abstract

**Background:**

Since the 1990s, community-associated methicillin resistant *staphylococcus aureus* (CA-MRSA) has emerged as an important global cause of skin and soft tissue infections. Little is known about the epidemiology of this pathogen in the Middle East.

**Methods:**

We conducted a prospective observational study in a single large teaching hospital in Dubai to identify the incidence of community-acquired methicillin resistant *staphylococcus aureus* (MRSA) among ambulatory patients presenting with purulent skin and soft tissue infections. We performed wound cultures and administered standard questionnaires to 100 cases presenting to the emergency department. Bivariate and multivariate analyses were performed to identify risk factors for MSRA versus other pathogens.

**Results:**

The prevalence of MRSA was 23% (18/78) among 78 culture-positive isolates and 29% (18/62) among Staphylococcus-positive isolates. 74% received antibiotics of which 4/74 (5%) received antibiotics appropriate for CA-MRSA infections. Multivariate adjusted analysis identified playing contact sports (OR 5.9 [95% CI 1.3–27.1]) and female sex (OR 6.3 [95% CI 1.6–24.8]) as independent risks for MRSA infection.

**Conclusions:**

This is the first study to describe the epidemiology of CA-MRSA in the ambulatory setting in the Middle East and demonstrates a substantial proportion of cases presenting with skin and soft tissue infections were CA-MRSA. Although most skin and soft tissue infections are abscesses for which the cornerstone of treatment is high quality incision and drainage, if adjunct antibiotics are prescribed in this setting, CA-MRSA-active antibiotics should be considered.

## Background

During the 1990s community-acquired MRSA (CA-MRSA) emerged in populations without exposure to health facilities and without traditional risk factors for MRSA [1, 2]. CA-MRSA infections exhibit different epidemiological characteristics, are resistant to fewer antibiotics, and express different toxin producing genes including, notably, the Panton-Valentine leucocidin a bicomponent toxin associated with recurrent skin and soft tissues infections (SSTIs) and necrotizing pneumonia [3].

CA-MRSA has emerged as a major global cause of purulent skin and soft tissues infections but little is known about the epidemiology of CA-MRSA in the Middle East [3–5] and no studies in the region have examined the prevalence of CA-MRSA in ambulatory settings. Given the absence of epidemiological data on this emerging pathogen we performed a cross-sectional observational study examining the prevalence of MRSA in purulent SSTI in ED patients in the United Arab Emirates (UAE). A secondary objective was to identify risk factors and clinical features of MRSA.

## Methods

From January 2011, we prospective enrolled a convenience sample of 100 adult patients presenting with purulent SSTIs to the emergency department (ED) of Rashid Hospital and Trauma Centre, a 599-bed public, tertiary-care, urban teaching hospital in Dubai that serves a largely poor migrant worker population. The annual ED census is ~ 140,000. Approximately 85–90% of the Dubai population are expatriates with the majority originating from the Indian subcontinent.

Prior to study enrollment, standardized study protocol training was conducted for ED physicians and nurses including enrollment criteria, questionnaire administration, and culture swab collection. After obtaining consent, enrolling clinicians collected standardized information on epidemiological and clinical features, potential risk factors, treatment and disposition (Table 1). Exclusion criteria included patients below the age of 18 years, hospitalization within the previous week, Bartholin, odontogenic or perianal abscesses, and infected post-operative incisional sites. We defined community-acquired MRSA according to United States Centers for Disease Control and Prevention criteria: (1) Positive culture for MRSA as an outpatient or within 48 h of hospital admission; (2) No medical devices or indwelling catheters that are permanently placed though the skin; (3) No history of MRSA infections; and (4) No recent history of hospitalization or residence in nursing home or long-term care facility. [6] Antibiotic sensitivity testing was performed using standard MIC and disk diffusion RH sensitivity methods.

**Table 1:** Univariate risk factors for MRSA infection versus other bacterial pathogens among emergency department patients with purulent skin and soft tissue infections

Variable	Sub-variable	MRSA (*n* = 18)	%	Other bacteria (*n* = 60)	%	Odds Ratio (95% CI)
Sex	Female	8	44%	12	20%	3.2 (1.0–9.9)
Nationality	Emirati national^a^	5	28%	14	23%	1.3 (0.4–4.2)
Accommodation	Shared	6	33%	19	32%	REF
	Home	9	50%	29	48%	1.0 (0.3–3.3)
	Camp	2	11%	9	15%	1.4 (0.2–8.5)
Past Medical History^b^	Yes	1	6%	9	15%	0.3 (0.0–3.8)
Risk factors	Incarcerated in past year	1	6%	0	0%	
	Play contact sports	5	28%	8	13%	2.5 (0.7–8.9)
	HH member with skin infection	1	6%	2	3%	0.6 (0.2–2.3)
	Recent hospitalization^c^	1	6%	3	5%	0.4 (0.1–5.9)
	ICU stay in past year	0	0%	0	0%	
Recent skin infection	Abscess/cellulitis	3	17%	15	25%	0.6 (0.2–2.4)
Antibiotic use last 3 months	Yes	5	28%	19	32%	1.3 (0.5–4.0)
MRSA suspected by clinician	Yes (vs no or not sure)	2	11%	2	3%	3.6 (0.5–27.3)
Current infection	Multiple abscesses/lesions	4	22%	4	7%	3.9 (0.9–17.7)
	Associated cellulitis	15	83%	48	80%	1.5 (0.4–6.0)

^a^Versus expatriates

^b^Included diabetes, hypertension, asthma, thalassemia, coronary artery disease, osteosarcoma. Some with multiple medical problems

^c^Past 3 months

*HH* Household

Crude and adjusted analyses using logistical regression to identify variables associated with MRSA versus other bacterial isolates were performed using IBM SPSS (V 19). A multivariate model using backward stepwise elimination was performed using all risk variables. Significance assumes *p* < 0.05 or an Odds Ratio (OR) that does not cross one.

### Ethics approval and consent to participate

All study participants provided written informed consent to participate in this study. The Medical Research Committee of the Dubai Health Authority, UAE, approved this study (October 2010).

## Results

Between January 2011 and June 2012, 99 patients with purulent SSTI infections were enrolled in the study and provided 100 culture samples (one patient with recurrent SSTI provided two specimen cultures 6 weeks apart); 75 (75%) were male with a median age of 30 (range 16–64); 74 (74%) were expatriates and 26% Emirati nationals. A bacterial culture isolate was identified in 78 patients including 44 methicillin-sensitive *Staphylococcus aureus* (SSSA) isolates and 18 MRSA isolates; other organisms were isolated from 16 patients. No organism was identified in 22 patients. When considering all patients enrolled, MRSA was isolated in 18 of 100 cases (18%); when considering only positive isolates, 18 of 78 (23%) were MRSA-positive. Of 62 total *S. aureus* isolates, 18 (29%) were MRSA positive. Seventeen of 18 MRSA cases (94%) met the criteria for CA-MRSA (one case had been hospitalized < 3-months prior to enrollment). Potential risk factors stratified by MRSA versus other bacteria isolates are listed in Table 1.

Multivariate adjusted analysis identified female sex (OR 6.3 [95% CI 1.6–24.8]) and playing contact sports (OR 5.9 [95% CI 1.3–27.1]) as independent risks for MRSA infection. A total of 74 patients (74%) received antibiotics of which 4/74 (5%) were antibiotics routinely recommended for CA-MRSA infections including clindamycin (3) and doxycycline (1). All of the 18 MRSA isolates were susceptible to trimethoprim-sulfamethoxazole in addition to vancomycin and linezolid. Isolates were not routinely tested for clindamycin or erythromycin susceptibility. There was no difference in the proportion of MRSA-positive cases versus non-MRSA positive cases requiring hospitalization (11/18, 61% versus 36/60, 60%, *p* = 0.33).

## Discussion

We present the first study in the Middle East to describe the epidemiological features of CA-MRSA among ambulatory patients presenting with SSTI. The overall prevalence of MRSA was 18% and the prevalence of MRSA among *S. aureus* isolates was 29%. The CA-MRSA prevalence reported in this study, although not as high as the United States, [7, 8] are similar to those recently reported in a multi-centre European study in which an average of 15% of SSTIs presenting to EDs were CA-MRSA, although substantial geographic variability was reported [9]. A number of studies have examined molecular characteristics of CA-MRSA in the UAE and Middle East or the prevalence of MRSA among laboratory *S. aureus* isolates [10–16] but no regional study has described the prevalence and epidemiology of MRSA among ambulatory patients.

Our study identified female sex and playing contact sports as independent risk factors for MRSA infection. Although playing contact sports is a well described risk factors for CA-MRSA in other settings, the association of female sex with MRSA was unexpected and requires further study. The risk of CA-MRSA versus other bacterial isolate was not different between the local Emirate and expatriate populations. Other risk factors were not identified, perhaps due to the relatively small sample size of this study or due to widespread community transmission of CA-MRSA beyond narrow epidemiological niches, which makes the identification of specific risk factors more challenging. Clinicians were not able to discriminate between MRSA and non-MRSA infections in this study.

Although most SSTI cases were treated with antibiotics, only a small proportion were treated with CA-MRSA-active antibiotics, a finding that is consistent with anecdotal observations that awareness of CA-MRSA as an important cause of purulent SSTI among healthcare providers is low. A substantial and likely increasing proportion of patients in this setting are likely to be infected with MRSA that will not respond to treatment with beta-lactam antibiotics including cephalosporins and combination or newer generation penicillins (for example amoxicillin-clavulanic acid). If antibiotic treatment is being considered as primary or adjunct treatment for purulent SSTI due to CA-MRSA, the Infectious Disease Society of American (IDSA) recommend clindamycin, trimethoprim-sulfamethoxazole, tetracyclines (minocycline, doxycycline) or Linazolid.

Historically, the management of purulent SSTI, particularly abscesses, has been incision and drainage, regardless of whether or not the responsible organism is MRSA. Recent data from the US, however, indicates that treatment with trimethoprim-sulfamethoxazole, which is active against most CA-MRSA strains, in addition to incision and drainage, improves cure rates in ED patients [7]. In that study, however, MRSA prevalence rates were ~ 45% raising questions about whether trimethoprim-sulfamethoxazole should be prescribed to patients with purulent SSTIs in countries where CA-MRSA prevalence rates may be lower. Regardless, if antibiotics are being considered in addition to standard incision and drainage, clinicians should be aware that in this setting one in four patients presenting to an ED with a purulent SSTI may be infected with CA-MRSA.

Limitations of this study include the small sample size and the longer-than expected enrollment period suggesting cases may have been missed and potentially risking selection bias. No molecular typing of samples was performed which would provide additional insight into the circulating strains and molecular epidemiology of CA-MRSA in the UAE.

## Conclusion

Given the global CA-MRSA epidemic will continue to evolve and given the UAE and the Middle East region will likely experience increasing rates of CA-MRSA-related disease in the future, clinicians should be prepared for and comfortable with managing SSTIs and other related conditions.

## Abbreviations

CA-MRSA: Community-associated methicillin-resistant *staphylococcus aureus*
ED: Emergency department
IDSA: Infectious Disease Society of American
SSTI: Skin and soft tissue infection
